# Association Between Missed Nursing Care and Nurse Fatigue: A Cross-Sectional Correlational Study

**DOI:** 10.3390/nursrep15080298

**Published:** 2025-08-13

**Authors:** Bushra Alshammari, Ghady Saud Alsaleh, Awatif Alrasheeday, Nadiah Baghdadi, Nabat Almalki, Farhan Alshammari, Amira Assiry, Mawahib Almalki

**Affiliations:** 1 Medical Surgical Nursing Department, College of Nursing, University of Hail, Hail 2440, Saudi Arabia; bu.alshammari@uoh.edu.sa; 2 Diabetes Center, King Fahed Hospital, Madinah Health Cluster, Madinah 42351, Saudi Arabia; gsalsaleh@moh.gov.sa; 3 Nursing Administration Department, College of Nursing, University of Hail, Hail 2440, Saudi Arabia; a.alrasheeday@uoh.edu.sa; 4 Nursing Management and Education Department, College of Nursing, Princess Nourah bint Abdulrahman University, Riyadh P.O. Box 84428, Saudi Arabia; nabaghdadi@pnu.edu.sa; 5 Nursing Department, Prince Sultan Military College of Health Sciences, Dhahran 34313, Saudi Arabia; njalmalki@psmchs.edu.sa; 6 Department of Pharmaceutics, College of Pharmacy, University of Hail, Hail 2440, Saudi Arabia; frh.alshammari@uoh.edu.sa; 7 King Faisal Medical City, Aseer Health Cluster, Abha 62527, Saudi Arabia; aaasiri@kfmcity.med.sa; 8 School of Nursing and Midwifery, Queen’s University Belfast, Medical Biology Centre, Belfast BT9 7BL, UK

**Keywords:** missed nursing care, nursing care/standards, occupational fatigue, work schedule tolerance, nurse staff, patient safety, hospital, Saudi Arabia, quality of healthcare, cross-sectional study

## Abstract

**Background**/**Objectives**: Missed nursing care—defined as any aspect of required patient care that is omitted or delayed—has emerged as a significant indicator of healthcare quality. Fatigue among nurses, particularly in high-demand environments, may contribute to care omissions. This study aimed to assess the prevalence and patterns of missed nursing care and its association with occupational fatigue among nurses working in Saudi hospitals. **Methods**: A cross-sectional correlational study was conducted among 183 registered nurses from multiple hospitals in the Hail and Madinah regions, Saudi Arabia. Data were collected using the Missed Nursing Care Scale (MISSCARE) and the Occupational Fatigue Exhaustion/Recovery Scale (OFER-15). Statistical analysis was performed to assess the relationships between missed care, fatigue, and demographic/work-related variables. **Results**: Nurses reported moderate levels of missed care, especially in basic care tasks such as oral hygiene, assistance with meals, and timely ambulation. The most frequently cited causes of missed care included insufficient staffing, high patient load, and a lack of support personnel. Occupational fatigue scores were also moderate, with notably low inter-shift recovery. A significant negative correlation was found between inter-shift recovery and missed care (r = −0.120, 95% CI: −0.23 to −0.005, *p* = 0.040), indicating that poorer recovery between shifts was associated with more frequent omissions. Other fatigue dimensions showed weak, non-significant associations with missed care. **Conclusions**: Missed nursing care is a prevalent issue in Saudi hospitals and is significantly influenced by organizational factors and nurses’ recovery between shifts. Interventions to improve staffing adequacy and promote rest and recovery may reduce care omissions and enhance patient outcomes.

## 1. Introduction

The delivery of safe, high-quality patient care is a fundamental obligation within modern healthcare systems. However, the phenomenon of missed nursing care—defined as any aspect of required patient care that is either omitted or significantly delayed—has emerged as a persistent concern in clinical practice [1]. Missed nursing care is widely recognized as a threat to patient safety, clinical outcomes, and overall healthcare quality. This multifaceted issue arises from a complex interplay of factors, including organizational constraints, workload demands, and staffing inadequacies, all of which challenge nurses’ ability to meet patient care expectations consistently and effectively [1,2].

Among the various contributors to missed nursing care, nurse fatigue has increasingly garnered attention as a salient factor. Fatigue in the nursing workforce encompasses a spectrum of physical, cognitive, and emotional exhaustion that impairs professional performance [3]. It is particularly pronounced in settings characterized by prolonged shifts, high patient acuity, and insufficient staffing, where the cumulative demands on nurses may exceed their capacity to recover adequately between shifts [3,4]. When fatigue prevails, nurses may experience diminished vigilance, impaired decision-making, and a reduced capacity to engage fully in patient care activities, thereby heightening the risk of missed care [5,6,7].

Globally, research has documented the prevalence and consequences of missed nursing care across diverse care environments. Studies have underscored the association between missed nursing care and adverse patient outcomes, such as medication errors, hospital-acquired infections, and an increased length of stay [8,9]. While some studies demonstrate a significant association between various dimensions of fatigue and missed nursing care [10,11,12], others suggest that the association becomes weaker or even non-significant once key organizational factors—such as staffing levels and workload—are accounted for. For instance, Crincoli et al. found that although both chronic fatigue and patient-to-nurse ratios were independently associated with missed care, nurse staffing levels had the most substantial impact, indicating that fatigue may play a secondary, indirect role in comparison to systemic factors [6].

In Saudi Arabia, the context of healthcare delivery presents unique challenges and opportunities for understanding this phenomenon [13]. Rapidly evolving healthcare reforms, rising patient volumes, and fluctuating workforce dynamics contribute to the complexity of nursing practice within the kingdom’s public and private healthcare institutions [14]. Understanding how nurse fatigue influences missed care in this context is essential for informing targeted interventions that promote nurse well-being and ensure optimal patient outcomes.

Recent studies conducted within Saudi Arabia underscore the relevance of missed nursing care and fatigue in the local context. For example, Al Muharraq et al. [15] found that the overall mean frequency of missed nursing care activities in Saudi hospitals was 1.37 on a 5-point scale, with ambulation and attending interdisciplinary care conferences being the most commonly omitted tasks. Additionally, Alshammari et al. [5] reported moderate to high levels of fatigue among emergency nurses in Saudi Arabia, influenced by workload, shift patterns, and departmental demands. These findings reflect a growing concern within Saudi healthcare institutions regarding the potential impact of nurse fatigue on care quality and safety.

National-level concerns about missed nursing care have also emerged, as reflected in Ministry of Health (MOH) reports and studies indicating variability in care quality across regions and institutions. For instance, a growing body of local evidence highlights missed fundamental tasks such as ambulation, discharge preparation, and patient education as recurring issues in Saudi hospitals [13,15]. Given the regional variability in hospital staffing and work environments, further investigations are warranted to explore the association between fatigue and missed nursing care across different regions of the kingdom.

This study therefore aims to explore the association between missed nursing care and nurse fatigue among registered nurses in Saudi Arabia. By examining the interplay between these critical factors within a diverse sample of nurses, the study seeks to provide evidence-based insights that can guide the development of organizational policies and clinical practices to mitigate missed nursing care and support the health and sustainability of the nursing workforce. It is hypothesized that higher levels of occupational fatigue are significantly associated with increased reports of missed nursing care.

### Theoretical Framework

This study is conceptually grounded in the Occupational Fatigue in Nursing model, which views nurse fatigue as a multidimensional phenomenon arising from the demands of the work system [16]. Steege and Pinekenstein [16] propose that all components of a nurse’s work environment—including staffing levels, workload intensity, work hours, and the physical and emotional demands of patient care—can generate a combination of physical, mental, and emotional stressors that contribute to fatigue [17]. Fatigue in this model is not a static condition but a dynamic state with acute (short-term, end-of-shift) and chronic (long-term, cumulative) dimensions. Critically, insufficient recovery between shifts allows acute fatigue to accumulate into chronic fatigue over time [18]. This multidimensional view aligns with Winwood et al.’s work on occupational fatigue, which differentiates acute fatigue, chronic fatigue, and inter-shift recovery as key elements of nurses’ fatigue experience (as measured by the Occupational Fatigue Exhaustion/Recovery Scale (OFER-15 scale)) [18].

In essence, when nurses face continuous high demands without adequate rest, their capacity for alertness and energy is progressively eroded. According to this framework, elevated fatigue levels can impair nurses’ performance and vigilance, thereby affecting care delivery. Fatigued nurses tend to be less alert, slower to respond, and have more difficulty concentrating and communicating effectively during patient care [17]. Research indicates that as fatigue accumulates, it negatively impacts work performance and increases the risk of errors or omissions [16].

Steege and Pinekenstein’s model explicitly acknowledges that acute and chronic fatigue can “affect every aspect of the nursing process” and ultimately influence patient and organizational outcomes [16]. In the context of missed nursing care, this means that a nurse who is physically and mentally exhausted may unintentionally omit or delay certain care tasks—not due to lack of knowledge or diligence, but because fatigue diminishes the nurse’s functional ability to carry out all required duties. Thus, drawing on this theoretical perspective, the present study examines how nurses’ acute fatigue, chronic fatigue, and inter-shift recovery relate to the incidence of missed nursing care, under the premise that improving recovery and managing fatigue could help reduce care omissions.

## 2. Materials and Methods

### 2.1. Design

This study employed a cross-sectional, correlational design to examine the association between missed nursing care and nurse fatigue among registered nurses. Data collection was conducted over a three-month period from February to May 2025.

### 2.2. Sample and Sampling

A non-probabilistic convenience sampling approach was employed to recruit participants for this study. The target population consisted of registered nurses working in five hospitals located in the cities of Hail and Almadinah, Saudi Arabia. The inclusion criteria were as follows: (1) registered nurses providing direct patient care and (2) consenting to participate in the study. The exclusion criteria included (1) nurses with less than one year of professional experience, (2) nurses in administrative positions without direct patient care responsibilities, and (3) nursing interns and students. This sampling strategy aimed to ensure that participants possessed relevant clinical experience and could provide insights into the relationship between missed nursing care and nurse fatigue in these healthcare settings.

An a priori power analysis was conducted using GPower version 3.1. To detect a small correlation (r = 0.20) with 80% power at a significance level of 0.05, a minimum of 193 participants was required. The achieved sample size of 183 was slightly below this target but still provides reasonable statistical power to detect correlations of small to moderate effect sizes, which aligns with the study objectives.

### 2.3. Setting

This study was conducted in five governmental hospitals located in two cities within the Kingdom of Saudi Arabia: Almadinah and Hail. In Almadinah, data were collected from Almadinah Almunawarah Hospital, Ohud Hospital, and King Fahd Hospital. In Hail, data collection took place in King Salman Specialist Hospital and King Khalid Hospital. These hospitals are publicly funded institutions that provide a broad spectrum of healthcare services to the local populations, free of charge. As integral components of the national healthcare system, these hospitals serve diverse patient populations and offer a range of inpatient and outpatient services across various medical and surgical specialties. This setting provided an appropriate and representative context to explore the relationship between missed nursing care and nurse fatigue among registered nurses working in diverse clinical environments.

### 2.4. Instruments

A structured, self-administered questionnaire was utilized in this study to collect comprehensive data from registered nurses. The first part of the questionnaire focused on collecting demographic and work-related information, including age, gender, marital status, nationality, level of education, department or unit of employment, shift type (fixed or rotating), and the average number of patients assigned per shift. This information provided essential context for understanding the characteristics of the nursing workforce and potential confounding variables.

The second part of the questionnaire incorporated the OFER-15 Scale, originally developed by Winwood and colleagues [3,19]. This scale comprises 15 items designed to assess three key dimensions of work-related fatigue: acute fatigue, chronic fatigue, and inter-shift recovery. Each item is rated on a 7-point Likert scale ranging from 0 (strongly disagree) to 6 (strongly agree). Each subscale comprises five items, yielding a maximum raw score of 30 per dimension, and a cumulative total of 90 points for overall fatigue. The OFER-15 is scored by calculating subscale scores for each of the three domains. Items 9, 10, 11, 13, and 15 are reverse coded to ensure the accurate measurement of fatigue and recovery constructs. The final scores for each subscale are then standardized on a 0–100 scale. Higher scores on the Acute and Chronic Fatigue subscales reflect greater levels of fatigue, while higher scores on the Inter-shift Recovery subscale indicate more effective recovery between shifts, reflecting lower cumulative fatigue. The OFER-15 scale has demonstrated strong psychometric properties, establishing it as a reliable and valid tool for assessing occupational fatigue in nursing populations. In the current sample, the scale showed high internal consistency, with an overall Cronbach’s alpha of 0.85.

The third part of the questionnaire featured the Missed Nursing Care Scale (MISSCARE), developed by Kalisch et al. [1]. This tool comprehensively measures both the frequency of missed nursing care activities and the underlying reasons for such omissions. The first section of the MISSCARE scale consists of 25 items evaluating the frequency with which nurses miss specific care activities, including elements of basic care, assessments, interventions, and monitoring. Each item is rated on a 5-point Likert scale (1 = never missed to 5 = always missed). For analysis, responses were dichotomized, with ratings of 1 and 2 categorized as care not missed, and ratings of 3 to 5 classified as care missed, thus facilitating the calculation of the prevalence of missed care activities. The classification reflects a commonly used threshold to simplify interpretation and enable meaningful comparison across settings. This approach follows the method adopted by Mainz et al. [20] and others who used the MISSCARE Survey in hospital contexts.

The second section of the MISSCARE scale includes 22 items designed to assess the potential reasons for missed nursing care. This section addresses a range of factors such as staffing adequacy, communication issues, material resources, and unexpected increases in patient volume or acuity. Each item is rated on a 4-point scale (1 = not a reason to 4 = significant reason), enabling the identification of prominent contributors to care omissions within the work environment. The MISSCARE tool has consistently demonstrated strong psychometric performance, with a Cronbach’s alpha of 0.892 reported in recent studies [21]. In the current study, internal consistency reliability was confirmed using Cronbach’s alpha. The MISSCARE Scale showed high reliability with α = 0.91 for Part A (missed care items) and α = 0.89 for Part B (reasons for missed care).

### 2.5. Recruitment Procedures

Data collection was carried out over a six-week period. The electronic survey was designed and disseminated using Google Forms to ensure efficient and accessible data gathering. A convenience sampling approach was used to recruit participants. To optimize the coverage of eligible nurses, the survey link was distributed to the head nurses of each participating hospital in Hail and Almadinah. The head nurses were responsible for sharing the survey link with their respective nursing staff, thus facilitating access across various departments and shift patterns.

To minimize bias associated with convenience sampling, clear inclusion and exclusion criteria were consistently applied to ensure comparability among participants. Nurses were recruited from multiple hospitals and various clinical departments (e.g., emergency, ICU, medical-surgical, pediatrics) to enhance representativeness. An anonymous online questionnaire link was distributed through official communication channels, such as institutional email, to reduce selection bias and encourage honest responses, thereby minimizing response bias.

Prior to participating, all potential participants received comprehensive written information about the study, including its objectives, the voluntary nature of participation, the confidentiality of their data, and the anonymity of their responses. The completion and submission of the online survey were considered as implied informed consent to participate in the study. Additionally, participants were reminded of their right to decline participation or withdraw from the study at any point without any adverse consequences.

### 2.6. Data Analysis

Data were analyzed using the Statistical Package for the Social Sciences (SPSS) version 28.0. Descriptive statistics, including frequencies, percentages, means, and standard deviations, were calculated to summarize the demographic and work-related characteristics of the study participants, as well as the scores for missed nursing care and fatigue subscales.

To examine the relationship between missed nursing care and nurse fatigue, correlation analyses were conducted using Spearman’s rank-order correlation coefficient (ρ). This approach was chosen to determine the strength and direction of linear relationships between continuous variables, specifically the subscale scores of missed nursing care and the three dimensions of fatigue measured by the OFER-15 scale.

The normality of continuous variables (missed nursing care scores) was assessed using the Shapiro–Wilk test, and non-normal distributions were confirmed (*p* ≤ 0.05). Accordingly, the Mann–Whitney U test was applied for comparisons between two groups, and the Kruskal–Wallis test was used for comparisons involving three or more groups.

A significance level of *p* < 0.05 was set for all inferential statistical tests.

### 2.7. Ethical Considerations

Ethical approval was obtained from the Research Ethics Committee of the University of Hail (Approval No.: H-2025-617; Date of Approval: 17 February 2025). The study was conducted in accordance with the ethical principles of the Declaration of Helsinki (1975), as revised in 2013. The study adhered to all applicable national and institutional ethical standards. Participants were fully informed about the purpose of the research, the voluntary nature of participation, data confidentiality, and their right to withdraw at any time without penalty. Data protection procedures were carefully implemented. The survey was anonymous and did not collect any identifiable personal information. Data were securely stored in password-protected files accessible only to the research team.

## 3. Results

Of approximately 400 nurses invited to participate, 183 completed the survey, yielding a participation rate of 45.75%. As shown in Table 1, the majority of respondents were over 30 years of age (73.2%), while 26.8% were aged 30 years or younger. Most participants were female (87.4%) and Saudi nationals (96.7%). In terms of marital status, more than half of the nurses were married (56.3%), followed by single (35.5%) and divorced or widowed individuals (8.2%).

With regard to educational attainment, 59.6% of participants held a bachelor’s degree, 26.2% held a diploma, and 14.2% had completed postgraduate studies. The distribution of professional experience was nearly even, with 49.7% reporting six years or less of work experience and 50.3% reporting more than six years.

Participants were employed across various clinical departments. The highest proportions were reported in the emergency room (ER) (20.8%), pediatric units (16.4%), and intensive care units (12.0%). Other departments included burn and trauma units (11.5%), medical-surgical units (10.9%), dialysis units (6.6%), obstetrics and gynecology (3.3%), and oncology units (3.3%). Additionally, 15.3% of nurses were assigned to other units.

In terms of work shifts, more than half of the participants worked day shifts (55.7%), while 38.3% worked rotating shifts and 6.0% worked night shifts exclusively. Regarding patient workload, 32.8% of nurses reported being assigned 10 or more patients per shift, while 30.6% were responsible for 1–3 patients. A further 25.1% and 11.5% reported caring for 4–6 and 7–9 patients per shift, respectively.

Participants were asked to report the frequency with which they missed various nursing care activities. As presented in Table 2, several fundamental aspects of patient care were reported as being missed with moderate frequency.

The highest mean scores were observed for mouth care (M = 3.20, SD = 1.40), setting up meals for patients who feed themselves (M = 3.10, SD = 1.30), feeding patients while the food is still warm (M = 3.00, SD = 1.40), patient bathing and skin care (M = 3.00, SD = 1.40), and attending interdisciplinary care conferences (M = 3.00, SD = 1.30). These findings suggest that aspects of hygiene, nutritional support, and care coordination were among the more frequently missed activities.

Several other essential tasks were also reported as commonly missed, including ambulation or mobilization three times per day (M = 2.80, SD = 1.30), assistive toileting within 5 min of request (M = 2.80, SD = 1.40), and turning patients every two hours (M = 2.90, SD = 1.40). Additionally, responding to call lights (M = 2.70, SD = 1.40) and hand hygiene practices (M = 2.70, SD = 1.40) were identified as being missed with moderate frequency.

On the other hand, tasks with slightly lower reported mean scores—suggesting less frequent omission—included vital sign assessments (M = 2.20, SD = 1.30), the full documentation of necessary data (M = 2.30, SD = 1.40), bedside glucose monitoring (M = 2.30, SD = 1.40), and IV or central line site care and assessments (M = 2.40, SD = 1.30). While these clinical responsibilities were less frequently missed, the scores indicate that they are not consistently performed in all shifts.

Overall, the findings highlight a pattern of missed care that spans both basic physical needs (e.g., hygiene, nutrition, mobility) and more technical or time-sensitive clinical interventions. These omissions may have significant implications for patient safety, satisfaction, and recovery outcomes.

Participants rated 22 potential factors contributing to missed nursing care using a 4-point Likert scale. As presented in Table 3, the most prominent reason cited was an inadequate number of staff, with the highest mean score of 3.5 (SD = 0.9) indicating a strong perceived influence on care omissions. This was closely followed by an inadequate number of assistive or clerical personnel (M = 3.4, SD = 1.0), heavy admission and discharge activity (M = 3.3, SD = 1.0), unexpected increases in patient volume or acuity (M = 3.3, SD = 1.0), and a lack of backup support from team members (M = 3.3, SD = 0.9).

Other frequently reported factors included unbalanced patient assignments (M = 3.2, SD = 1.0), emotional or physical exhaustion (M = 3.2, SD = 1.0), tension within the nursing team (M = 3.2, SD = 1.0), and inadequate leadership support (M = 3.2, SD = 1.0). Communication issues with ancillary departments (M = 3.1, SD = 1.0), multitasking demands (M = 3.1, SD = 1.0), and an unavailability of supplies or equipment (M = 3.1, SD = 1.0) also emerged as relevant contributors.

Several factors received comparatively lower ratings but were still considered meaningful. These included medications being unavailable when needed (M = 2.9, SD = 1.0), supplies or equipment not functioning properly (M = 2.9, SD = 1.0), and a lack of cues or reminders (M = 2.9, SD = 1.0). Additionally, inadequate hand-offs (M = 2.8, SD = 1.0), caregivers being off-unit or unavailable (M = 2.8, SD = 1.1), and the failure of nursing assistants to report unprovided care (M = 2.9, SD = 1.0) were rated moderately.

### Occupational Fatigue Scores (OFER-15)

The occupational fatigue of participants was assessed using the OFER-15 scale, which measures three distinct domains: acute fatigue, chronic fatigue, and inter-shift recovery. Each domain comprises five items scored on a 0–6 Likert scale, with total raw scores ranging from 0 to 25 per subscale. Scores were also converted into percentage values out of 100 to facilitate interpretation and comparison across dimensions.

As shown in Table 4, the mean score for acute fatigue was 14.9 (SD = 7.2), corresponding to a mean percentage of 49.8%, suggesting that participants experienced moderate levels of fatigue that developed over the course of a shift. Similarly, the mean score for chronic fatigue was 14.6 (SD = 5.7), translating to a mean percentage of 48.6%, which reflects a sustained and cumulative type of fatigue across multiple shifts or longer durations.

The mean score for inter-shift recovery was 14.3 (SD = 3.7), equivalent to 47.7%, indicating suboptimal recovery between shifts. This finding suggests that nurses may not be regaining sufficient physical and psychological energy before returning to work, which can potentially exacerbate acute and chronic fatigue.

The overall fatigue score—calculated as the sum of the three subscales—had a mean of 43.8 (SD = 13.5) out of a possible 90, corresponding to a mean percentage of 48.7%. This cumulative value reflects a moderate level of occupational fatigue among the participants, with considerable variability noted across individuals (range: 20.0 to 75.0).

As presented in Table 5, the correlations between missed nursing care and acute fatigue (r = −0.056, 95% CI: −0.20 to 0.09, *p* = 0.452), chronic fatigue (r = −0.055, 95% CI: −0.19 to 0.09, *p* = 0.456), and overall fatigue (r = −0.056, 95% CI: −0.20 to 0.09, *p* = 0.452) were weak and not statistically significant. However, a statistically significant negative correlation was found between inter-shift recovery and missed nursing care (r = −0.120, 95% CI: −0.23 to −0.005, *p* = 0.040). This indicates that lower levels of inter-shift recovery were significantly associated with higher reports of missed nursing care.

There were no statistically significant differences in missed nursing care scores by age group (*p* = 0.828), gender (*p* = 0.970), nationality (*p* = 0.531), years of experience (*p* = 0.742), marital status (*p* = 0.594), educational level (*p* = 0.273), department (*p* = 0.747), shift type (*p* = 0.226), or nurse-to-patient ratio (*p* = 0.557). This suggests that perceived missed care did not significantly differ across these categories.

In relation to occupational fatigue, a statistically significant difference was observed between male and female nurses (*p* = 0.034), with female nurses reporting higher levels of occupational fatigue (Mean Rank = 95.15) compared to their male counterparts (Mean Rank = 70.07). No statistically significant differences in occupational fatigue were found based on age (*p* = 0.956), nationality (*p* = 0.189), experience (*p* = 0.487), marital status (*p* = 0.439), educational level (*p* = 0.907), department (*p* = 0.502), shift type (*p* = 0.395), or nurse-to-patient ratio (*p* = 0.716) (see Table 6).

## 4. Discussion

This study identified a moderate prevalence of missed nursing care among nurses in Saudi hospitals, particularly in fundamental tasks such as oral care, timely feeding, and patient mobilization. Occupational fatigue levels were also moderate, with insufficient inter-shift recovery emerging as a significant factor associated with care omissions. These findings highlight the interplay between workload, nurse well-being, and the delivery of essential patient care within the Saudi healthcare context.

Critical nursing interventions like regular ambulation, turning immobile patients, a prompt response to call lights, and assistive toileting were also missed at moderate rates. In contrast, technical tasks (e.g., vital sign monitoring, glucose checks, IV site care) were missed less often, though still not consistently performed each shift. This pattern suggests that omissions tend to cluster around basic care and comfort measures, echoing prior findings that fundamental nursing care—mobility, hygiene, nutrition, and patient education/discharge planning—is especially vulnerable when resources are strained. Such tasks may be perceived as lower priority under pressure, yet their neglect can adversely impact patient outcomes (e.g., increased infection risk, discomfort, or delayed recovery).

Consistent with the global literature, our participants identified staffing shortages and high workload as the dominant reasons for missed care. The highest-rated factors contributing to missed care were an inadequate number of nursing staff and support personnel, heavy admissions/discharges, unexpected surges in patient acuity, and a lack of teamwork backup. These align with numerous international studies that pinpoint human resource inadequacy as a principal driver of care omissions [22,23]. For instance, a large Saudi study in Jazan found that missed nursing care was “mainly caused by human resource shortage” [15], while a recent Ethiopian study reported a 62.5% overall missed care rate that was significantly linked to resource availability and teamwork problems [24].

Our findings reinforce that when nurse-to-patient ratios are stretched and support is lacking, nurses are forced to triage duties, often deferring or omitting non-urgent but important care aspects. We observed that about one-third of our nurses were caring for 10 or more patients per shift, a workload likely incompatible with comprehensive care delivery. This aligns with evidence that a high patient load is a critical risk factor for missed care: a 2024 U.S. study of night-shift nurses showed that high patient-to-nurse ratios had the strongest effect on missed care, even more than individual fatigue levels [6].

In our study, nearly all the top-cited reasons for missed care (unbalanced assignments, sudden influx of patients, multitasking, etc.) reflect work system issues rather than a lack of nurse effort or knowledge. It is notable that communication breakdowns and insufficient leadership support were also moderately high on the list of reasons, suggesting that organizational climate factors (such as teamwork, inter-department cooperation, and manager support) play a role in enabling nurses to complete their care. These insights underscore that missed care is a systemic problem requiring administrative attention to staffing levels, workflow, and team communication, rather than being solely attributable to individual nurses.

### 4.1. Nurse Fatigue and Its Association with Missed Care

A central focus of this study was the relationship between nurse fatigue and missed care. Overall, participating nurses reported moderate levels of occupational fatigue on the OFER-15 scale. The mean scores for acute fatigue (within a shift) and chronic fatigue (cumulative overtime) were approximately 50% of the maximum, indicating that many nurses felt noticeably tired during and across shifts, while inter-shift recovery scores were also suboptimal, suggesting that nurses often did not fully recuperate between work days. These fatigue levels are concerning yet not surprising given the demands reported—for example, over half of our sample worked either rotating shifts or permanent nights, schedules known to disrupt circadian rhythm and recovery. Indeed, a Korean study found that “unhealthy” scheduling characterized by long hours and a lack of rest was linked to more frequent missed nursing care [25]. Proper rest between shifts emerged as a pivotal factor in our analysis as well: inter-shift recovery had a significant inverse correlation with missed care. In other words, nurses who were better able to recover between shifts reported fewer care omissions, whereas those coming to work still fatigued were more likely to miss tasks. However, it is important to note that the observed correlation (r = −0.12) represents a small effect size despite reaching statistical significance (*p* = 0.040). This suggests that while inter-shift recovery contributes to missed nursing care, its impact is modest and likely interacts with broader organizational and staffing factors. Future studies with larger sample sizes are needed to confirm and further explore this relationship.

This finding resonates with the theoretical model by Winwood et al. [19], which posits that insufficient recovery (along with acute and chronic fatigue) depletes the physical and cognitive reserves nurses need for vigilant care delivery. It also aligns with recent evidence from U.S. studies of hospital night nurses, where high chronic fatigue and low inter-shift recovery were each associated with more missed care [6,26]. Notably, in Crincoli’s study, chronic fatigue remained an independent predictor of missed care even when controlling for staffing, although adequate staffing had the largest impact. The data from the present study showed only a weak, non-significant trend between higher chronic or acute fatigue and missed care frequency. One reason for this discrepancy could be the generally moderate fatigue levels in our sample or the inclusion of day-shift nurses (who typically report slightly less fatigue than night nurses), which may not have been sufficient to produce measurable effects on care delivery. By contrast, studies focusing on high-stress contexts or night shifts exclusively (like Crincoli et al. [6]) have documented a clearer fatigue–missed care link when fatigue reaches more extreme levels.

Another explanation is that nurses may prioritize critical tasks even when fatigued, resulting in fatigue having a diffuse impact on the overall frequency of missed care. For example, a fatigued nurse might still ensure medications and vital signs are checked (to avoid immediate harm) but may let less urgent care aspects lapse (like ambulation or oral care). This task prioritization could reduce the linear correlation in a general ward context and mask the more subtle effects of fatigue on basic care omissions. Evidence suggests that in high-pressure or understaffed environments, nurses often focus their limited energy on clinically critical and time-sensitive tasks, such as medication administration and vital sign monitoring, while deferring or omitting basic but essential care activities like oral hygiene, ambulation, or feeding [27,28]. This task-triaging behavior may attenuate the measurable relationship between acute or chronic fatigue and overall missed care frequency. Additionally, systemic factors—particularly staffing adequacy and workload—may have a stronger mediating influence, overshadowing the contribution of individual fatigue dimensions. This aligns with previous studies showing that once staffing and workload are accounted for, the direct relationship between fatigue and missed care can weaken or become non-significant [6].

Interestingly, only the recovery aspect of fatigue showed a significant relationship with missed care in our results, suggesting that the ability to rest and recharge between shifts is crucial. Nurses who cannot adequately recover likely accumulate sleep debt and exhaustion, which impairs concentration, vigilance, and motivation [29]. The current study result is congruent with recent research highlighting quick shift turnarounds and a lack of time off as predictors of errors and omissions—for instance, short inter-shift intervals (<11 h) have been shown to impede recovery and increase fatigue-related lapses in performance [30]. Thus, ensuring sufficient rest periods and avoiding excessively long shifts or quick returns could be an actionable strategy to reduce missed care incidences related to fatigue.

It is also worth noting that nurse burnout and mental exhaustion have been linked to missed care in other studies, underlining the broader impact of caregiver well-being on care quality. For example, a post-pandemic survey in Thailand found that for each unit increase in nurses’ emotional exhaustion (burnout), the odds of reporting missed nursing care increased by 61% [31]. Many of our participants self-reported symptoms like feeling “emotionally or physically exhausted” as a reason for missing care. While we did not formally measure burnout in this study, the overlap between fatigue and burnout (specifically exhaustion) is recognized; prolonged fatigue states can evolve into burnout. Our results, in concert with the literature, imply that interventions to reduce nurse fatigue (through better schedules, adequate breaks, and supportive work environments) may also mitigate burnout and thereby decrease missed care. Indeed, nurses in our study who had better inter-shift recovery may have been protected from missing care, just as those with lower burnout have shown better care outcomes in other research. Future studies could explicitly measure burnout alongside fatigue to further elucidate these relationships in the Saudi context.

### 4.2. Interpretation in Light of the Conceptual Framework

The findings of this study align closely with the Occupational Fatigue in Nursing model, which conceptualizes fatigue as a dynamic and multidimensional phenomenon shaped by the demands of the work system. The moderate levels of acute and chronic fatigue observed suggest that nurses experience both shift-based and cumulative exhaustion, while the significant association between inter-shift recovery and missed care supports the model’s emphasis on the importance of adequate recuperation between shifts.

The patterns of missed care identified—particularly omissions in basic nursing tasks such as hygiene, feeding, and mobility—further reinforce the model’s premise that when cognitive and physical resources are depleted, nurses tend to prioritize urgent, high-risk interventions (e.g., medication administration, vital signs) at the expense of fundamental care. Additionally, the influence of systemic factors, such as inadequate staffing and heavy patient load, reflects the model’s recognition that organizational structures play a central role in generating or mitigating fatigue. This interplay underscores the need for interventions that address both individual nurse recovery and broader systemic conditions to reduce missed nursing care.

### 4.3. Comparison with Other Studies

Our study adds to a growing body of evidence on missed nursing care internationally and within Saudi Arabia. The overall prevalence and pattern of missed care we observed are generally in line with global reports, though some differences emerged. The average frequency of missed care in our sample was at a “sometimes” level for multiple activities, which appears higher than what one large Saudi survey reported. That nationwide study by Al Muharraq et al. [15] noted that although missed care exists in Saudi hospitals, it was significantly lower than international rates and largely concentrated in tasks like attending care conferences and patient ambulation. In contrast, our participants (from Hail and Madinah regions) reported moderately frequent misses in direct care activities like hygiene and feeding. This could reflect regional or setting differences—perhaps the hospitals we sampled face greater staffing pressures or higher patient acuity. It might also result from data collection differences; nonetheless, it signals that some Saudi facilities experience missed care levels comparable to those in Western studies. Internationally, studies in the US, Europe, and Asia have consistently found that care omissions often involve basic care (e.g., mobilization, mouth care, patient education) and correlate strongly with nurse workload and staffing issues [6,27,28]. The present research reinforces those patterns in the Saudi context. For example, similar to our results, a Jordanian study found that nurses perceived a shortage of human resources as the top reason for missed care in medical–surgical units [32]. Likewise, a recent systematic review affirmed that inadequate nurse staffing is the most frequently cited cause of missed or “unfinished” nursing care globally [33]. Encouragingly, the Saudi study by Al Muharraq et al. [15] suggested that Saudi Arabia’s overall missed care rates were lower than elsewhere, possibly due to ongoing improvements in staffing and quality initiatives. Our data, however, indicate that missed care is still a salient issue at least in some Saudi institutions, warranting continued vigilance.

A growing body of research has established a connection between nurse fatigue and the incidence of missed nursing care. For example, Crincoli et al. [6] reported that night-shift nurses in the United States who experienced higher levels of chronic fatigue were significantly more likely to omit required nursing tasks. Similarly, a recent study from South Korea found that nurses with greater fatigue and poorer sleep quality were more likely to report missed or “left undone” care, particularly under high workload conditions [10]. In our study, only inter-shift recovery showed a statistically significant—though modest—correlation with missed care. This suggests that the relationship between fatigue and missed care may not always be immediately evident in general settings but becomes more pronounced when fatigue accumulates over time or is left unaddressed. These findings underscore the need to shift focus from isolated episodes of tiredness to broader, long-term patterns of fatigue and recuperation. Accordingly, we recommend that future research in Saudi Arabia investigate this dynamic using longitudinal designs and objective tools, such as actigraphic sleep monitoring or validated fatigue scales, to assess how fluctuations in nurse fatigue influence care quality over time.

By contrast, others have found clear connections; our discussion already noted that Crincoli et al. [6] linked high chronic fatigue with more missed tasks in the U.S. Another study from South Korea similarly documented that nurses with greater fatigue (and poorer sleep) tend to report more instances of missed or “left undone” care, especially under high workload conditions [10]. This finding that only inter-shift recovery had a significant (albeit small) correlation with missed care suggests that the linkage may be subtle in a general setting but becomes pronounced when fatigue accumulates or goes unrelieved. This underscores the importance of looking beyond just acute tiredness on a given shift to the broader pattern of fatigue and recuperation over time. In short, our results contribute to this debate by highlighting inter-shift recovery as a key piece of the puzzle. We recommend that future research in Saudi Arabia explores this further, perhaps using longitudinal designs or objective fatigue measures (such as actigraphic sleep data or validated fatigue scoring) to see how fluctuations in nurse fatigue translate to care quality over time.

Another noteworthy comparison point is that we found no significant differences in missed care reporting across most nurse demographics (age, experience, education, etc.). Both younger and older nurses, novice and veteran, reported similar levels of missed care. This aligns with some studies that suggest that missed care is a system-level issue affecting nurses broadly, rather than a problem isolated to less experienced staff [33]. However, other studies have observed differences—for example, one report noted that nurses with under 5 years experience missed more care, particularly in fundamental tasks [20]. It is possible that in our sample, strong teamwork or mentoring in the hospitals helped even out the performance between junior and senior nurses. Alternatively, the nearly even split of experience in our sample and our relatively moderate sample size (N = 183) may have limited our ability to detect subtle differences.

Similarly, we did not observe significant variations in the frequency of missed care by shift type (day vs. night vs. rotating) or by unit type. While one might expect night shift or certain high-acuity units (e.g., ICU) to have more missed care due to lower staffing at night or higher patient needs, our data did not show a statistically clear pattern. This could again be due to sample distribution (only 11 full-time night nurses participated, which may be too few to draw firm conclusions). It contrasts a bit with studies focused on night shifts, which often report a higher incidence of missed care at night owing to skeletal staffing and nurse fatigue [6]. The lack of a detectable difference in our study may actually be a positive sign—it could indicate that the participating hospitals maintain relatively consistent care standards across shifts, or that rotating shift systems distribute the load. Nonetheless, this is an area that merits further investigation, as targeted interventions might be needed for night shift nurses who face circadian disruptions and typically fewer resources.

### 4.4. Implications for Practice and Policy in Saudi Arabia

The study findings carry important implications for nursing management and healthcare policy, especially within Saudi Arabia’s rapidly evolving healthcare system. First and foremost is the critical need to address nurse staffing and workload. The fact that “inadequate number of staff” was the top-rated reason for missed care, and the fact that tasks were missed most frequently when nurses had 10 or more patients, sends a clear signal: ensuring safe nurse-to-patient ratios should be a policy priority. Evidence consistently demonstrates that safe nurse-to-patient ratios are critical in reducing care omissions and improving patient outcomes [6,8,28]. A systematic review by Chiappinotto et al. [33] further underscores that inadequate staffing and heavy workloads are the most frequent antecedents of missed nursing care across healthcare systems. Implementing fatigue risk management systems, including regulated work hours and mandated rest breaks, has been recommended to reduce occupational fatigue and its downstream effects on patient safety [17,29]. Furthermore, enhancing ancillary staff support and fostering positive team communication are recognized strategies to prevent basic care omissions such as feeding and hygiene, which are often deprioritized under high workload conditions [27].

Saudi Arabia’s MOH has recognized this challenge; recent reports indicate aggressive recruitment efforts, with the nursing workforce expanding by tens of thousands in the past few years [14]. These efforts are aligned with the kingdom’s Vision 2030 initiatives to improve healthcare quality. The study underscores that simply adding headcount is not enough—the distribution and skill of staff mix must also match patient acuity and the demand for care in each unit. Hospital administrators should regularly assess workload indicators (e.g., nurse-to-patient assignments, overtime hours, missed care reports) as quality metrics. Flexible staffing strategies, such as float pools or adjusted nurse assignments based on real-time patient acuity, could help buffer against the sudden surges in volume that our respondents cited as leading to missed care. Additionally, bolstering support staff (nurse aides, clerks, transporters) is crucial, as nurses noted that a lack of assistive personnel directly contributed to missed care. Adequate ancillary support frees registered nurses to focus on skilled care tasks and prevents basics like feeding and hygiene from being overlooked due to time constraints.

Another implication is the need to promote nurse well-being and reduce fatigue through better scheduling and rest opportunities. While nursing is inherently demanding, evidence-based scheduling can mitigate avoidable fatigue. We recommend that hospitals consider limiting excessive consecutive working days and night shifts, ensuring nurses have sufficient hours off between shifts (at least the 11 h minimum recommended in some jurisdictions to prevent quick returns) [30], and enforcing reasonable limits on overtime. Rotating shift patterns should be designed if possible to rotate forward (day–evening–night) rather than backward, as forward rotation is kinder on circadian adjustment. Evidence from this study that inter-shift recovery is linked to missed care highlights that managers should pay attention to how roster patterns allow for recovery. Strategies like arranging longer breaks after a string of shifts, offering nap breaks during long night shifts, or using self-scheduling with guidelines can improve nurses’ rest and satisfaction [29].

Furthermore, given that female nurses in our study reported significantly higher fatigue than males, employers should be mindful of potential gender-specific stressors. The nursing workforce in Saudi Arabia (as reflected in our sample, ~87% female) often includes many working mothers or those juggling family responsibilities. Prior research indicates female nurses experience higher fatigue partly due to work–family conflicts and societal expectations, whereas male nurses more often cite long shifts or lack of support as fatigue drivers. Thus, family-friendly policies (like flexible schedules or on-site childcare services) and promoting a supportive work culture can particularly help our predominantly female nursing workforce manage fatigue. Ultimately, investing in nurse well-being is not just a staff satisfaction issue but a patient safety imperative, as fatigued nurses are more prone to errors and omissions.

From a clinical practice standpoint, another implication is to strengthen team communication and leadership support on the units. Participants noted that poor teamwork or leadership contributed to missed care (e.g., lack of backup help, tension in the team, inadequate supervision). Nurse managers and charge nurses should foster an environment where staff feel comfortable to voice when they are overwhelmed and where asking for help is encouraged. Implementing brief daily huddles or using team-based nursing models may help redistribute workload on the fly so that essential care tasks are covered even on busy shifts. Additionally, streamlining workflows and reducing non-value-added tasks can free up time for patient care. For instance, ensuring supplies are readily available (addressing the issue of equipment/supply unavailability cited by nurses) and leveraging health assistants for clerical duties can reduce the cognitive load and fatigue on nurses. Hospitals could also implement missed care monitoring as part of quality improvement programs—e.g., regularly surveying staff on missed care or auditing patient records for signs of care omissions—to identify trouble spots. When patterns are found (such as mouth care or patient turning often missed), targeted interventions like checklists, reminder systems, or dedicated “turn teams” might be employed.

Finally, at the policy level within Saudi Arabia, our study’s insights support integrating nurse fatigue management into patient safety and quality initiatives. The MOH and healthcare accreditation bodies might develop guidelines or standards that address nurse work hours, rest breaks, and staffing ratios explicitly as quality criteria. Just as policies exist for physician duty hours, similar attention to nursing work hours could be beneficial. Additionally, given the finding that missed nursing care did not vary significantly by nurse characteristics, it suggests that solutions must be system wide. Approaches such as hiring additional nurses, offering resilience training, and providing mental health support for nurses could all be part of a comprehensive strategy. Importantly, tackling the root causes of missed care (staffing, workflow, and fatigue) will not only improve patient outcomes but also nurse outcomes—research has shown missed care is linked to nurse job dissatisfaction and intent to leave. Indeed, the Jazan study found nurses’ turnover intention was a significant predictor of missed care, implying a vicious cycle. By breaking this cycle—through supportive policies, adequate staffing, and attention to nurse recovery—Saudi healthcare leaders can improve care quality and retain a healthier, more motivated nursing workforce.

### 4.5. Strengths and Limitations

This study contributes valuable local data on missed care and nurse fatigue in Saudi Arabia, a context where such research has been relatively limited. By using validated instruments (MISSCARE survey and OFER-15 fatigue scale) and including multiple hospitals from two regions, we gained a nuanced picture of how workload and fatigue interplay to affect care delivery. The response rate and sample size (N = 183) were reasonable for an exploratory correlational study, and the diversity of departments strengthens the generalizability of our findings across different clinical settings.

However, we acknowledge several limitations. The use of self-reported questionnaires may introduce response bias; nurses might under- or over-report missed care and fatigue due to recall issues or social desirability. Additionally, the collection of all data through self-reported measures in a single survey session may introduce common-method bias, potentially inflating the observed associations due to shared method variance. Future research should consider using multiple data sources or temporally separating measurement of variables to reduce this risk.

Objective measures (like actual staffing data, patient outcomes, or physiological fatigue measures) were not collected and would add depth in future studies. The cross-sectional design also limits causal inference—while we found associations (e.g., poor recovery linked with more missed care), we cannot definitively say fatigue causes missed care or vice versa. It is conceivable, for instance, that struggling to keep up with care tasks contributes to nurses feeling exhausted (a reverse effect). Longitudinal research could clarify temporal relationships.

Additionally, the study did not systematically follow the CHERRIES (Checklist for Reporting Results of Internet E-Surveys) guidelines during the design and reporting of the online questionnaire, which may affect the transparency and standardization of some methodological aspects. For instance, a common concern in online surveys is the possibility that a single respondent may submit multiple entries. These methodological omissions may introduce uncertainties in data integrity and limit the replicability of the study in future research.

Another limitation is that our sample, while covering two cities, may not represent all Saudi hospitals. Healthcare facilities in other regions or the private sector might have different challenges. Additionally, some subgroup analyses (like shift type differences) had unequal group sizes, reducing power to detect differences. We also did not account for certain potential confounders such as exact shift length, overtime hours, or specific patient acuity levels, which could influence both fatigue and missed care.

### 4.6. Future Research Directions

This study opens several avenues for future research. One important direction is to investigate interventions to reduce missed care—for example, testing whether implementing a fatigue management program (adjusted schedules, rest breaks, fatigue awareness training) in a hospital can lead to measurable reductions in care omissions and improvements in nurse outcomes. Another area is to explore the role of recovery more deeply: qualitative studies could interview nurses about how they rest between shifts and what barriers they face in achieving adequate recovery (childcare, second jobs, etc.), especially in Saudi Arabia’s cultural context.

Additionally, given that missed care is a multifaceted issue, mixed-methods research combining quantitative surveys with observations or interviews could provide richer insights into why certain tasks (like mouth care or patient turning) tend to be missed and how nurses make real-time decisions when juggling tasks under fatigue. Since our study did not find differences by experience or education, it would be worthwhile to examine whether targeted mentorship or continuing education could further reduce missed care, or if truly the key lies in system factors alone.

Intervention-based research should also be prioritized to test practical solutions. Implementing structured fatigue risk management programs—such as optimized shift scheduling, mandatory rest breaks, and fatigue awareness training—could be evaluated through cluster randomized controlled trials across multiple hospitals to determine their effectiveness in reducing nurse fatigue and minimizing missed nursing care.

Finally, expanding research to include patient perspectives and outcomes would be valuable—linking missed nursing care incidents to patient satisfaction, readmissions, or clinical outcomes in Saudi hospitals would underscore the tangible impact of the issue and build the business case for interventions. In line with global patient safety goals, Saudi Arabia can benefit from research that identifies effective strategies to ensure “zero missed care,” thereby safeguarding patient well-being and supporting the nurses who are the backbone of its healthcare system.

## 5. Conclusions

This study underscores that missed nursing care is a significant patient safety concern in Saudi hospitals and is closely intertwined with workforce factors, particularly nurse fatigue and insufficient recovery between shifts. When nurses are overextended and return to work without adequate rest, essential care tasks such as feeding, mobility, and hygiene are more likely to be delayed or omitted. Conversely, improving staffing adequacy and facilitating effective inter-shift recovery hold considerable potential to reduce care omissions and enhance patient outcomes.

A key takeaway from our findings is that missed nursing care is largely driven by systemic and organizational conditions rather than individual nurse performance. The high frequency of omissions linked to staffing shortages, heavy workloads, and a lack of support personnel highlights the need for structural solutions rather than placing responsibility solely on frontline nurses. Addressing these factors requires a coordinated effort at both the institutional and policy levels to ensure a sustainable nursing workforce and safe care delivery.

In light of these results, there is a compelling need for Saudi healthcare administrators and policymakers to integrate fatigue risk management into national nursing standards. Establishing safe nurse-to-patient ratios that reflect unit acuity, enforcing minimum rest periods between shifts to support inter-shift recovery, and ensuring adequate ancillary staff to cover basic care tasks are essential steps. Embedding the routine monitoring of missed nursing care within hospital quality improvement programs can further help identify patterns early and guide targeted interventions.

By adopting evidence-based staffing, scheduling, and support strategies, Saudi Arabia can reduce missed nursing care, safeguard patient safety, and strengthen nurse well-being. These measures align with the kingdom’s Vision 2030 healthcare objectives and should be considered a national priority. Ultimately, ensuring that nurses have both the resources and the recovery time necessary to deliver complete, high-quality care is not only a workforce issue but also imperative for patient safety.

## Figures and Tables

**Table 1:** Demographic and work-related characteristics of participants (N = 183).

Variable	Category	n (%)
Age	≤30 years	49 (26.8)
	>30 years	134 (73.2)
Gender	Male	23 (12.6)
	Female	160 (87.4)
Marital Status	Single	65 (35.5)
	Married	103 (56.3)
	Divorced/Widowed	15 (8.2)
Nationality	Saudi	177 (96.7)
	Non-Saudi	6 (3.3)
Educational Level	Diploma	48 (26.2)
	Bachelor’s degree	109 (59.6)
	Postgraduate	26 (14.2)
Years of Experience	≤6 years	91 (49.7)
	>6 years	92 (50.3)
Department/Unit	ER	38 (20.8)
	ICU	22 (12.0)
	Medical-Surgical Unit	20 (10.9)
	Pediatric Unit	30 (16.4)
	Burn and Trauma Unit	21 (11.5)
	Obstetrics/Gynecology Unit	6 (3.3)
	Dialysis Unit	12 (6.6)
	Oncology Unit	6 (3.3)
	Other	28 (15.3)
Shift Type	Day shift	102 (55.7)
	Night shift	11 (6.0)
	Rotating shifts	70 (38.3)
Number of Patients Assigned per Shift	1–3	56 (30.6)
	4–6	46 (25.1)
	7–9	21 (11.5)
	≥10	60 (32.8)

Emergency room = ER, Intensive Care Unit = ICU.

**Table 2:** Frequency of missed nursing care activities (N = 183).

Conceptual Categories	Nursing Care Item	Mean	SD
Basic Care Interventions	Ambulation/mobilization three times per day or as ordered	2.80	1.30
	Turning patient every 2 h	2.90	1.40
	Feeding patient when the food is still warm	3.00	1.40
	Setting up meals for patient who feeds themselves	3.10	1.30
	Assist with toileting needs within 5 min of request	2.80	1.40
	Skin/Wound care	2.50	1.30
	Patient bathing/skin care	3.00	1.40
	Mouth care	3.20	1.40
	Hand washing	2.70	1.40
Planning and Teaching	Patient teaching about illness, tests, and diagnostic studies	2.50	1.30
	Patient discharge planning and teaching	2.50	1.30
Assessment and Monitoring	Assess effectiveness of medications	2.60	1.30
	Bedside glucose monitoring as ordered	2.30	1.40
	Patient assessments performed each shift	2.50	1.30
	Focused reassessments according to patient condition	2.60	1.30
	IV/central line site care and assessments according to hospital policy	2.40	1.30
	Vital signs assessed as ordered	2.20	1.30
	Monitoring intake/output	2.40	1.30
	Full documentation of all necessary data	2.30	1.40
	Adequate surveillance of confused/impaired patients	2.60	1.30
Communication and Coordination	Response to call light initiated within 5 min	2.70	1.40
	PRN medication requests acted on within 15 min	2.60	1.40
	Medications administered within 30 min before or after scheduled time	2.60	1.40
	Emotional support to patient and/or family	2.60	1.30
Interventions Requiring Team Support	Attend interdisciplinary care conferences whenever held	3.00	1.30

**Table 3:** Reasons for missed nursing care (N = 183).

Item	Mean	SD
Was there an inadequate number of staff?	3.5	0.9
Were there urgent patient situations (e.g., a patient’s condition worsening)?	3.0	1.0
Was there an unexpected increase in patient volume and/or acuity on the unit?	3.3	1.0
Was there an inadequate number of assistive and/or clerical personnel?	3.4	1.0
Were patient assignments unbalanced?	3.2	1.0
Were medications unavailable when needed?	2.9	1.0
Was the hand-off from the previous shift or sending unit inadequate?	2.8	1.0
Did other departments fail to provide needed care?	3.0	1.0
Were supplies or equipment unavailable when needed?	3.1	1.0
Were supplies or equipment not functioning properly when needed?	2.9	1.0
Was there a lack of backup support from team members?	3.3	0.9
Was there tension or a communication breakdown with ancillary/support departments?	3.1	1.0
Was there tension or a communication breakdown within the nursing team?	3.2	1.0
Was there tension or a communication breakdown with the medical staff?	3.0	1.1
Did the nursing assistant fail to report that care was not provided?	2.9	1.0
Was the caregiver off the unit or unavailable?	2.8	1.1
Was there heavy admission and discharge activity?	3.3	1.0
Did emotional or physical exhaustion occur?	3.2	1.0
Was there inadequate supervision of nursing assistants?	3.0	1.1
Were there interruptions or multitasking demands?	3.1	1.0
Was there a lack of cues or reminders?	2.9	1.0
Was support from leadership inadequate?	3.2	1.0

**Table 4:** Distribution of Scores for occupational fatigue.

	Mean	SD	Minimum	Maximum	Mean %
Acute Fatigue	14.9	7.2	0.0	25.0	49.8
Chronic Fatigue	14.6	5.7	3.0	25.0	48.6
Inter-shift Recovery	14.3	3.7	7.0	25.0	47.7
Overall OFER	43.8	13.5	20.0	75.0	48.7

**Table 5:** Correlation between missed of nursing care with occupation fatigue.

Variable Pair	n	ρ (rho)	95% (C.I.)	*p* Value
Acute Fatigue (AF)	183	−0.056	−0.204 to 0.094	0.452
Chronic Fatigue (CF)	183	−0.055	−0.203 to 0.095	0.456
Inter-shift Recovery (IR)	183	−0.120	−0.23 to −0.005	0.040
Overall Fatigue	183	−0.056	−0.203 to 0.094	0.452

Note: ρ (rho) = Spearman’s correlation coefficient.

**Table 6:** Comparison of missed nursing care and occupational fatigue across demographic and work-related characteristics (N = 183).

Variable			Missed Nursing Care		Occupational Fatigue	
		N	Mean Rank	*p* Value	Mean Rank	*p* Value
Age	≤30 Years	49	90.59	0.828	91.64	0.956
	>30 Years	134	92.51		92.13	
Gender	Male	23	92.39	0.970	70.07	0.034 *
	Female	160	91.94		95.15	
Nationality	Saudi	177	92.45	0.531	92.95	0.189
	Non-Saudi	6	78.67		64.08	
Experience	≤6 Years	91	90.06	0.742	20.76	0.487
	>6 Years	92	91.74		23.43	
Marital Status	Single	65	90.14	0.594	85.42	0.439
	Married	103	94.81		96.19	
	Divorced/Widowed	15	80.80		91.73	
Educational Level	Diploma	48	82.73	0.273	94.86	0.907
	Bachelor	109	93.57		90.84	
	Postgraduate	26	102.54		91.56	
Department	ER	38	90.76	0.747	86.55	0.502
	ICU	22	100.07		101.55	
	Medical-Surgical Unit	20	94.33		93.20	
	Pediatric Unit	30	87.57		85.38	
	Burn and Trauma Unit	21	89.95		78.57	
	Obstetrics/Gynecology	6	130.58		84.58	
	Dialysis Unit	12	88.86		107.86	
	Oncology Unit	6	90.75		106.46	
	Other	28	70.42		72.08	
Shift Type	Day shift	102	95.90	0.226	92.75	0.395
	Night shift	11	106.55		71.09	
	Rotating shifts	70	84.03		94.20	
Number of Patients Assigned per Shift (Nurse-to-patient ratio)	1–3	56	64.83	0.557	85.47	0.716
	4–6	46	61.76		92.95	
	7–9	21	54.98		94.12	

A *p*-value < 0.05 was considered statistically significant. * indicates significant difference, Emergency room = ER, Intensive Care Unit = ICU.

## Data Availability

The data supporting the findings of this study are not publicly available due to ethical restrictions and the protection of participants’ confidentiality. However, de-identified data may be made available from the corresponding author upon reasonable request and with approval from the relevant institutional ethics committee.

## Public Involvement Statement

No public involvement in any aspect of this research.

## Guidelines and Standards Statement

This manuscript was drafted against the STROBE (Strengthening the Reporting of Observational Studies in Epidemiology) guidelines for cross-sectional observational research. The checklist was used to ensure comprehensive and transparent reporting.

## Abbreviations

The following abbreviations are used in this manuscript:

MOH	Ministry of Health
MISSCARE	Missed Nursing Care Survey
OFER-15	Occupational Fatigue Exhaustion/Recovery Scale (15-item version)
ER	Emergency room
CHERRIES	Checklist for Reporting Results of Internet E-Surveys
